# Ocular Loiasis in Morocco: A Case Report From a Non-endemic Area

**DOI:** 10.7759/cureus.110716

**Published:** 2026-06-12

**Authors:** Hala Ait Amar, Houda Safwate, Amine Razzak, Mohamed Bouazza, Mohamed Elbelhadji

**Affiliations:** 1 Department of Ophthalmology, Faculty of Medicine, Mohammed VI University of Health Sciences (UM6SS), Casablanca, MAR; 2 Ophthalmology, Cheikh Khalifa International University Hospital, Mohammed VI University of Sciences and Health (UM6SS), Casablanca, MAR

**Keywords:** calabar swelling, loa loa, non-endemic area, ocular loiasis, ocular parasitosis, subconjunctival worm

## Abstract

Loiasis is a parasitic infection caused by the filarial nematode *Loa loa*, endemic to Central and West Africa. Its occurrence in non-endemic regions such as Morocco is rare and may pose a diagnostic challenge. We report the case of a Moroccan patient presenting with an intermittent sensation of a mobile foreign body in the eye, associated with redness and photophobia. The patient reported no recent travel to endemic regions but had close contact with individuals originating from Cameroon, a loiasis-endemic area, raising questions regarding possible indirect or remote exposure. Following several consultations, spontaneous exteriorization of a subconjunctival worm was observed. The diagnosis was supported by clinical observation of the subconjunctival worm, blood parasitological testing demonstrating microfilaremia, and the presence of Calabar swellings. The patient was treated with ivermectin followed by albendazole, resulting in complete clinical recovery without recurrence during follow-up. This case highlights the importance of considering loiasis in the differential diagnosis of atypical ocular symptoms, even in non-endemic settings, and underscores the value of multidisciplinary collaboration for timely diagnosis and appropriate management.

## Introduction

Loiasis is a human filariasis caused by *Loa loa*, a parasite transmitted by deerflies of the genus *Chrysops* [1,2]. This parasitic disease mainly affects populations in Central and West Africa, where it remains a significant public health problem, with millions of individuals estimated to be at risk of infection [2,3]. Conversely, in non-endemic regions such as Morocco, cases remain exceptional, with only a few imported case reports described in the literature.

We present a rare case of ocular loiasis in a 45-year-old woman living in Morocco without recent travel history to endemic zones. However, close contact with neighbors originating from Cameroon, a loiasis-endemic country, was noted as a possible epidemiological context. The patient reported an intermittent sensation of a mobile foreign body in the eye, accompanied by redness and photophobia. Despite multiple consultations during which no worm was detected, detailed ophthalmologic examination ultimately led to the clinical observation of a mobile worm in the conjunctival sac. The diagnosis was supported by blood parasitological examination demonstrating microfilaremia, together with the presence of Calabar swellings.

This case highlights the diagnostic difficulties encountered in non-endemic countries, where this condition may easily be overlooked [4]. It also emphasizes the importance of including loiasis in the differential diagnosis of atypical ocular symptoms with a mobile character, even in the absence of recent travel to endemic areas, while considering possible remote or indirect exposure.

This report aims to raise clinicians’ awareness of this rare parasitosis, promote better clinical recognition and appropriate management in unusual epidemiological contexts, and highlight the importance of collaboration between ophthalmologists and infectious disease specialists.

## Case presentation

A 45-year-old woman, a resident of Morocco with no recent history of travel to endemic areas, consulted for intermittent discomfort in the right eye, described as a sensation of a mobile foreign body, associated with conjunctival redness and photophobia. The patient repeatedly reported a sensation of a moving foreign body in the eye, a symptom highly suggestive of subconjunctival migration of the parasite. Although she denied recent travel to Central or West Africa, close contact with neighbors originating from Cameroon, a loiasis-endemic country, was noted as a possible epidemiological context.

Visual acuity was preserved at 10/10 for distance vision, and intraocular pressure was within normal limits. Examination of ocular adnexa was unremarkable. No obvious subconjunctival worm was initially observed. The anterior chamber was quiet, the vitreous clear, and fundus examination showed no abnormalities.

**Figure 1:**
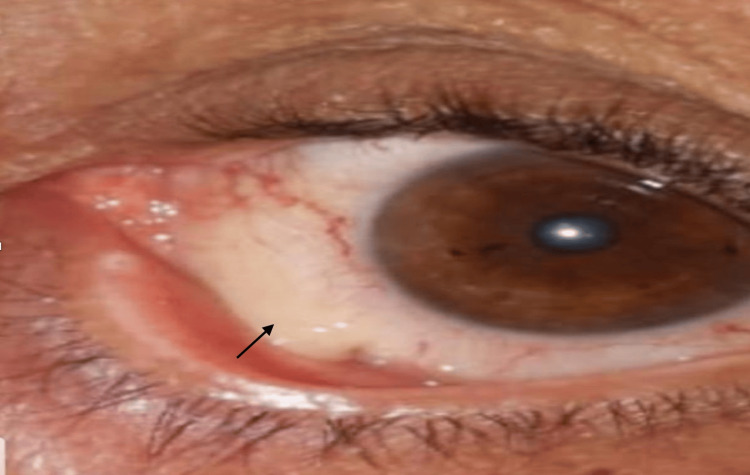
Subconjunctival migration of a mobile filiform worm in the right eye. The black arrow indicates the worm visible beneath the conjunctiva.

While waiting in the consultation area, the patient suddenly felt the parasite migrating (Figure 1) and manually extracted a filiform mobile worm from the conjunctival sac of the right eye.

**Figure 2:**
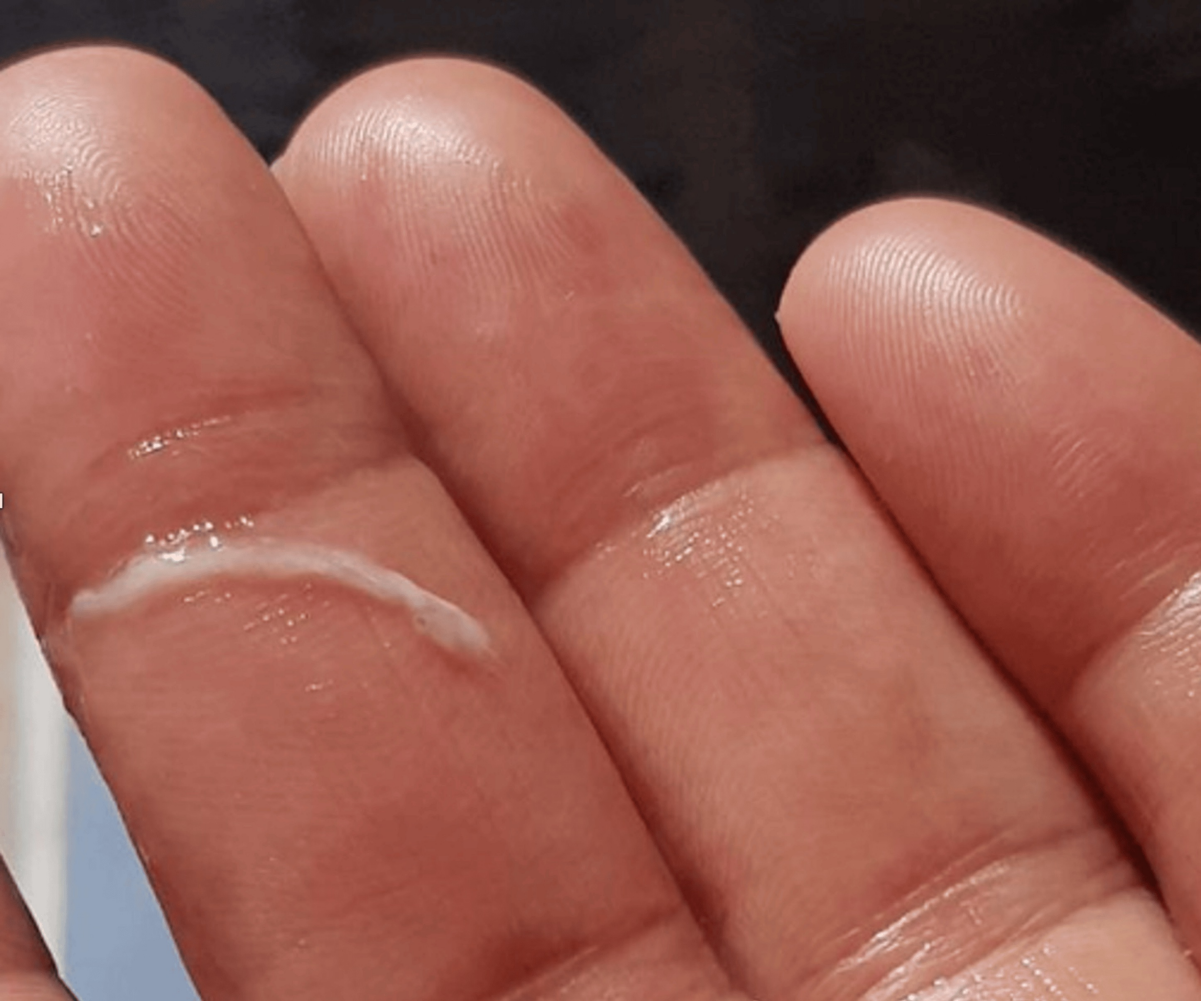
Mobile filiform worm immediately after spontaneous removal from the conjunctival sac of the right eye.

**Figure 3:**
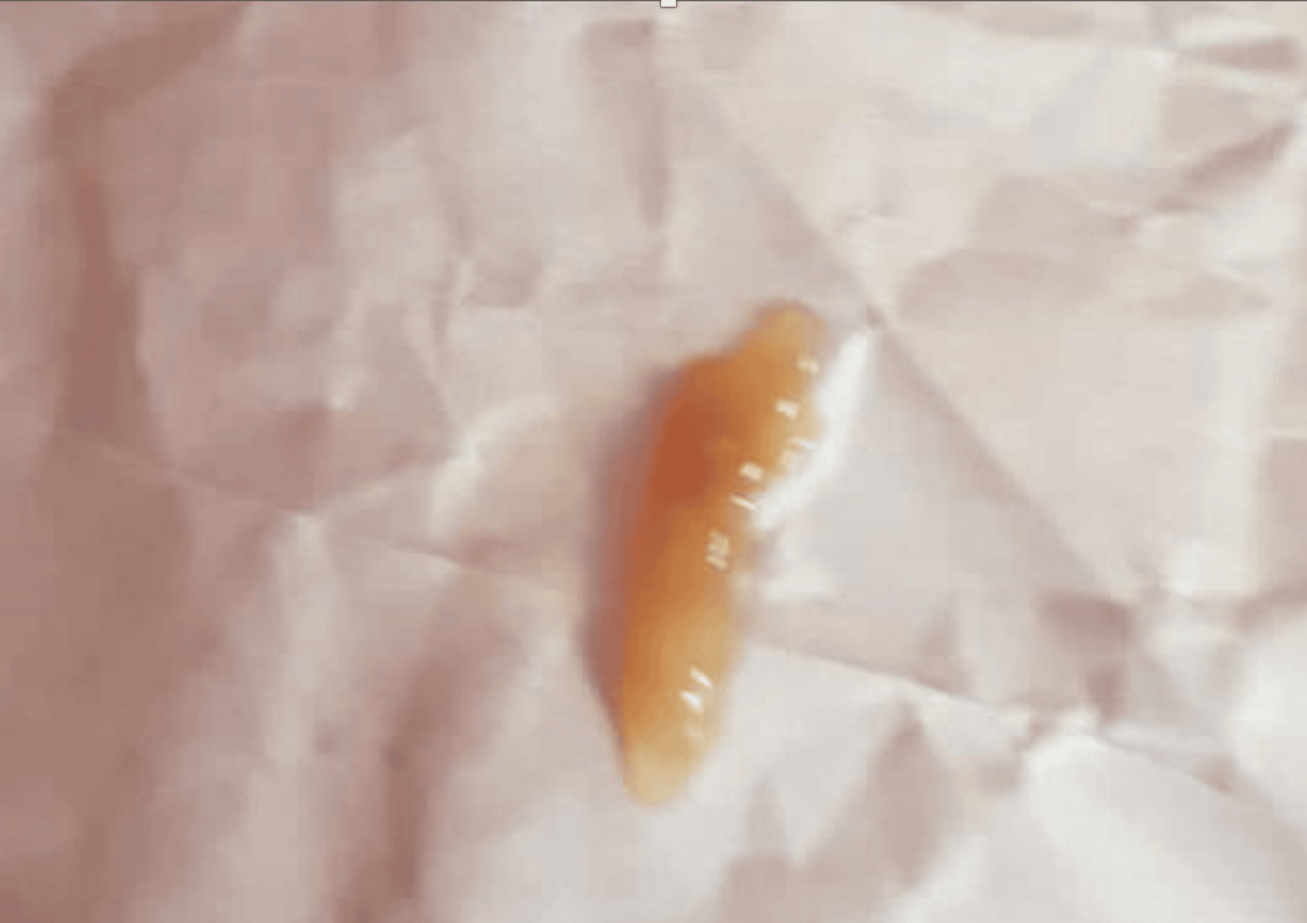
Close-up view of the mobile filiform worm showing its elongated morphology.

The parasite was immediately shown to the medical team for clinical examination (Figures 2, 3).

**Figure 4:**
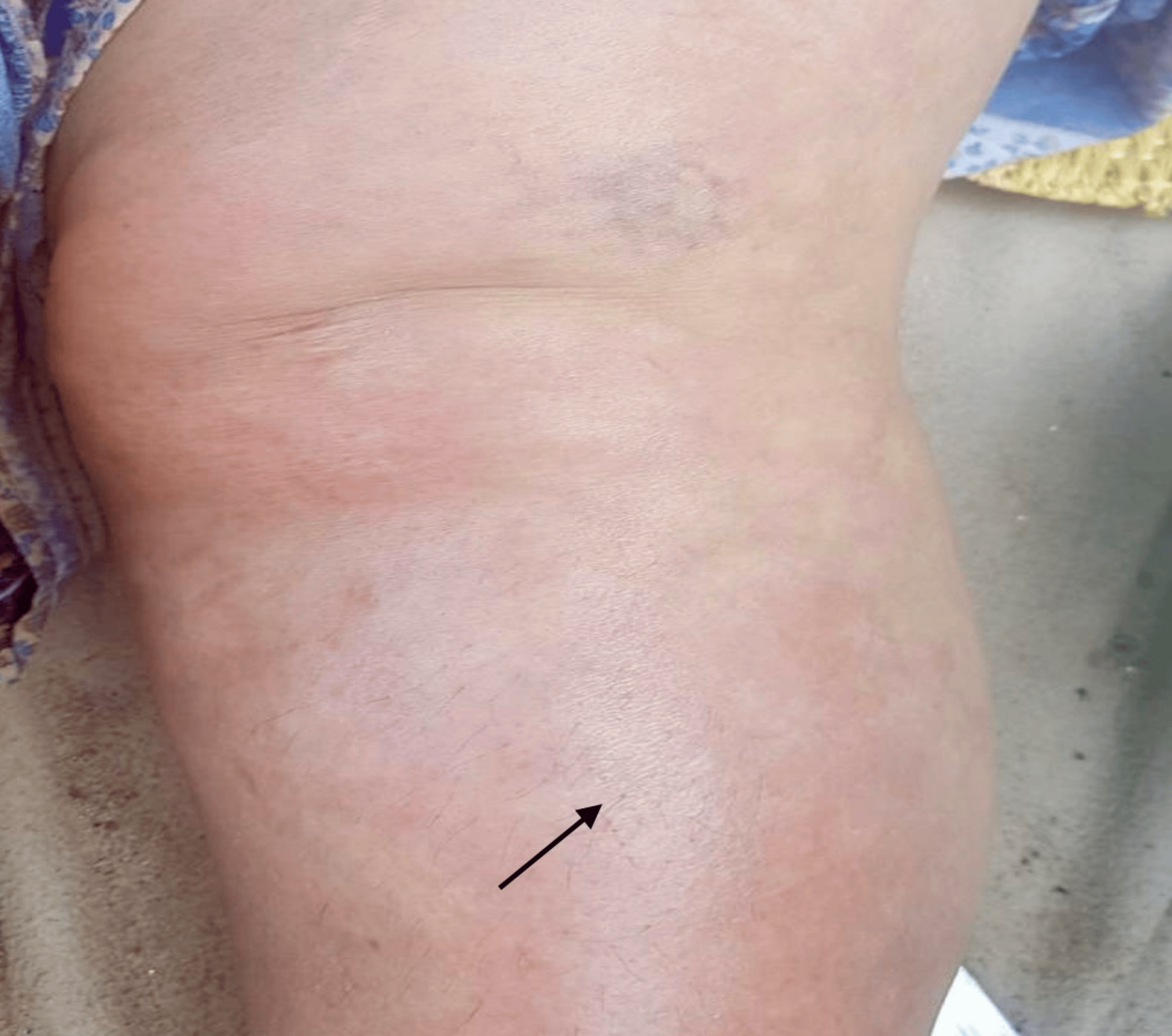
Calabar swelling at the knee, corresponding to transient localized subcutaneous edema. The black arrow indicates the edematous area.

Additionally, the patient exhibited intermittent limb swellings suggestive of Calabar swellings (Figure 4).

Laboratory investigations included a complete blood count, which revealed mild leukocytosis, lymphopenia, and thrombocytopenia, without eosinophilia. Blood parasitological examination demonstrated microfilaremia, supporting the diagnosis of loiasis in conjunction with the clinical findings and the presence of Calabar swellings.

The patient was treated with oral ivermectin (21 mg as a single dose), followed 15 days later by albendazole (400 mg twice daily for 15 days). Monthly follow-up showed complete clinical recovery, with no recurrence at three months.

## Discussion

Loiasis is a human filariasis caused by *Loa loa*, a nematode transmitted by the bite of deerflies of the genus *Chrysops* [1]. This parasitosis is strictly endemic to forested regions of Central and West Africa, notably Cameroon, Congo, Gabon, and Nigeria [2]. The adult parasite migrates through subcutaneous and conjunctival tissues, causing sometimes spectacular but often misleading symptoms [3].

Following inoculation, the larvae (microfilariae) require about one year to mature into adults. These adult worms can live for several years in connective tissue, migrating under the skin or within the eye, causing various symptoms. The appearance of a visible worm beneath the conjunctiva is one of the most specific signs, although relatively rare [4]. The average size of the worm ranges from 3 to 7 cm but can exceptionally reach 10 cm [5].

Clinically, ocular manifestations most often include conjunctival hyperemia, photophobia, tearing, and especially an intermittent sensation of a mobile foreign body, often described precisely by patients [6]. In our case, the patient repeatedly reported this sensation of mobility despite the absence of parasite visualization during the initial examinations.

Furthermore, transient subcutaneous swellings (Calabar swellings), usually painless, are characteristic. These swellings result from an immune reaction to the migration of adult worms in tissues [7]. They are strongly suggestive in a compatible clinical context, as observed in our patient.

Diagnostic confirmation relies on visualization of the migrating worm, detection of daytime microfilaremia, or molecular techniques when available [8,9]. In our patient, the diagnosis was supported by three concordant findings: clinical observation of a mobile subconjunctival worm, the presence of Calabar swellings, and blood parasitological examination demonstrating microfilaremia.

The differential diagnosis includes other causes of mobile ocular symptoms or subconjunctival parasites, such as ophthalmomyiasis, subconjunctival dirofilariasis, cysticercosis, inflammatory conjunctival lesions, and retained foreign bodies. The combination of a migrating subconjunctival worm, Calabar swellings, and confirmed microfilaremia strongly favored the diagnosis of loiasis.

Treatment aims to eliminate both the visible parasite and circulating microfilariae. Antiparasitic therapy must be adapted to the microfilarial burden because rapid parasite destruction may induce severe inflammatory reactions. In our patient, ivermectin followed by albendazole was selected according to established therapeutic recommendations, resulting in complete clinical recovery without recurrence during follow-up [10,11].

This case raises an important epidemiological question because the patient did not report recent travel to endemic regions. Although close contact with individuals originating from Cameroon was noted, the exact mode of acquisition remains uncertain and should be considered a limitation of this report. Remote exposure, undocumented travel, or other unrecognized epidemiological factors cannot be excluded.

Only a limited number of ocular loiasis cases have been reported in North Africa, highlighting the exceptional nature of this presentation in the region [3,12]. This rarity may delay diagnosis when clinicians are unfamiliar with the disease.

Thus, ophthalmologists should consider loiasis in the differential diagnosis of atypical ocular symptoms associated with a sensation of migration, even in non-endemic settings. Close collaboration between ophthalmologists, parasitologists, and infectious disease specialists remains essential for timely diagnosis and appropriate management.

## Conclusions

Ocular loiasis, though rare, remains a striking and diagnostic manifestation of *Loa loa*, particularly in endemic contexts or migration settings. This case underlines the importance of considering loiasis when faced with atypical ocular signs, especially when associated with a sensation of parasite migration, even outside historically endemic areas. Careful ophthalmologic examination, thorough clinical history, blood parasitological confirmation of microfilaremia, and multidisciplinary collaboration allow timely diagnosis and appropriate management. Recognizing this clinical presentation is crucial to avoid diagnostic errors and inappropriate treatments. This case also highlights the need to maintain parasitological vigilance in an era of increasing globalization and population mobility.
